# Turn off that night light! Light-at-night as a stressor for adolescents

**DOI:** 10.3389/fnins.2024.1451219

**Published:** 2024-07-31

**Authors:** Grace E. Guindon, Cloey A. Murphy, Maria E. Milano, Joseph A. Seggio

**Affiliations:** Department of Biological Sciences, Bridgewater State University, Bridgewater, MA, United States

**Keywords:** light, circadian, HPA axis, teenagers, clock genes, sleep

## Abstract

Light-at-night is known to produce a wide variety of behavioral outcomes including promoting anxiety, depression, hyperactivity, abnormal sociability, and learning and memory deficits. Unfortunately, we all live in a 24-h society where people are exposed to light-at-night or light pollution through night-shift work - the need for all-hours emergency services – as well as building and street-lights, making light-at-night exposure practically unavoidable. Additionally, the increase in screentime (tvs and smart devices) during the night also contributes to poorer sleep and behavioral impairments. Compounding these factors is the fact that adolescents tend to be “night owls” and prefer an evening chronotype compared to younger children and adults, so these teenagers will have a higher likelihood of being exposed to light-at-night. Making matters worse is the prevalence of high-school start times of 8 am or earlier – a combination of too early school start times, light exposure during the night, and preference for evening chronotypes is a recipe for reduced and poorer sleep, which can contribute to increased susceptibility for behavioral issues for this population. As such, this mini-review will show, using both human and rodent model studies, how light-at-night affects behavioral outcomes and stress responses, connecting photic signaling and the circadian timing system to the hypothalamic–pituitary adrenal axis. Additionally, this review will also demonstrate that adolescents are more likely to exhibit abnormal behavior in response to light-at-night due to changes in development and hormone regulation during this time period, as well as discuss potential interventions that can help mitigate these negative effects.

## 1 Introduction

One unfortunate here-to-stay part of modern life is exposure to light-at-night either through shift- work or exposure to artificial building lights and streetlights – light pollution – which can cause disruptions to the endogenous biological clock. The increased use of light-emitting devices during the night, something prevalent among adolescents, also contributes to light-at-night exposure. Current research in humans of both biological sexes has consistently shown that exposure to light-at-night leads to abnormal behavior, including anxiety (Paksarian et al., 2020), depression (Helbich et al., 2020), social issues (Deibel et al., 2020), and exacerbates the symptoms of existing psychiatric disorders (Esaki et al., 2022). Studies using nocturnal rodents have corroborated the anti-social, anxiogenic, and depression-inducing effects of light-at-night, similar to what was found in diurnal humans (Zhou et al., 2018; Michaud et al., 2022; Medeiros Contini and Seggio, 2023). Furthermore, the altered timing of light exposure disrupts melatonin secretion (a hormone that regulates sleep and its levels depressed by light-at-night) leading to increased cortisol during the night (Rahman et al., 2019). These changes in mood due to light-at-night can be attributed to poorer sleep and increased stress responses. Therefore, minimizing light-at-night exposure is essential for preserving mental health and reducing the risk of the development of psychiatric disorders, particularly among adolescents, which exhibit increased evening preference and sensitivity to light-at-night.

### 1.1 The circadian timing system and connections to the HPA axis

**Figure 1:**
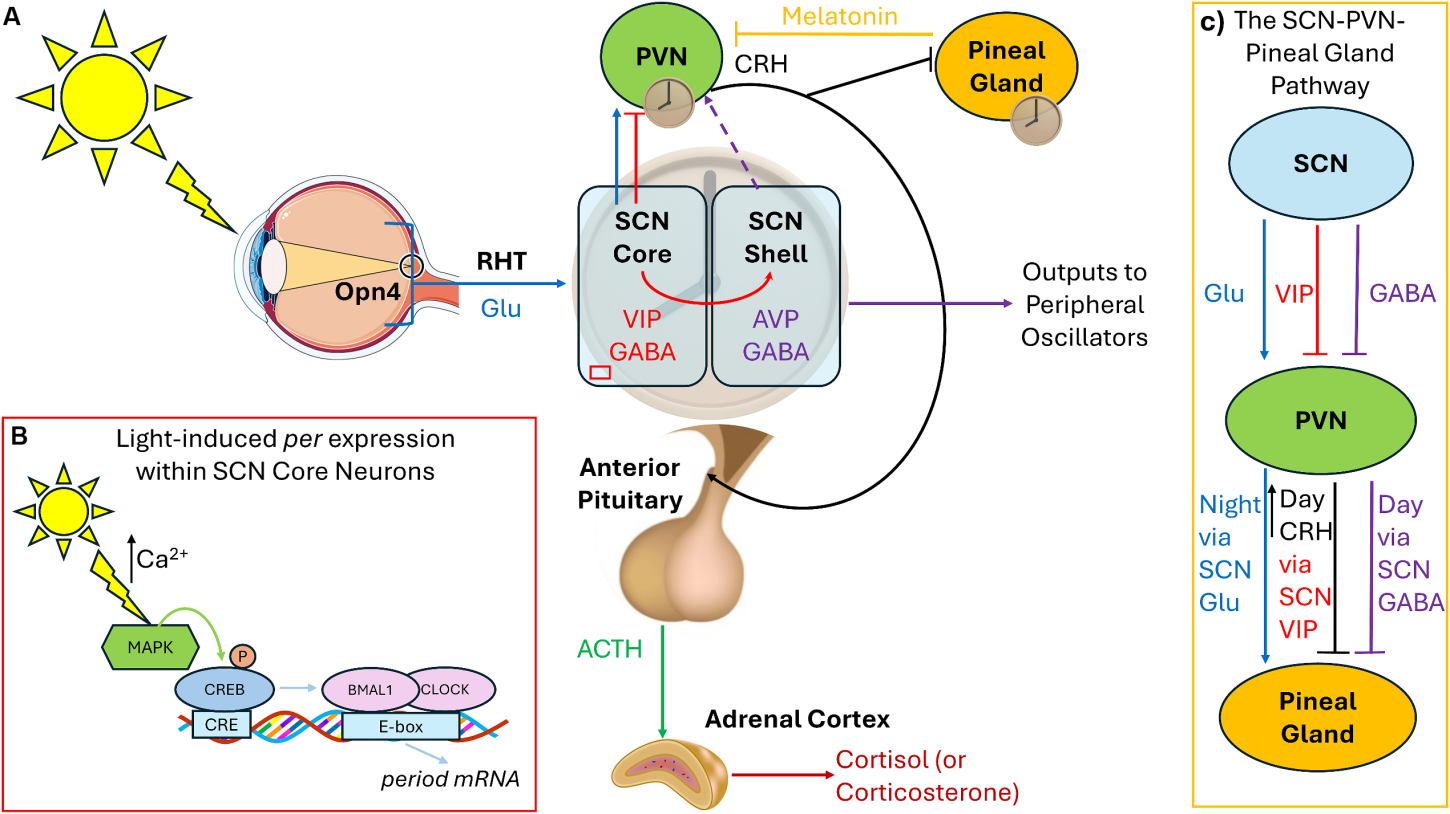
Connections among the circadian pacemaker, pineal gland, and HPA axis. **(A)** Under standard light:dark cycles, the SCN receives photic signals through the RHT via specialized Opn4-containing retinal ganglion cells, which conveys glutamatergic signals to the SCN core (blue). The SCN core contains VIP-containing neurons, which express circadian clock genes in response to light input. The SCN shell contains AVP-containing cells, receives no retinal photic input, but receives GABA and VIP signaling from the core (red). GABAergic signals from the SCN shell inhibit the PVN, and AVP is used as outputs to synchronize peripheral oscillators (purple). Direct glutamate and VIP projections from the SCN core to the hypothalamic PVN also exist. Hypophysiotropic neurons within the PVN release CRH (black) activating the anterior pituitary to release ACTH (green) to modulate the adrenal gland to release cortisol/corticosterone. AVP outputs exert control over the HPA axis by regulating CRH, depending upon whether the animal is nocturnal (inhibition) or diurnal (stimulation), leading to release of cort at the beginning of the active phase (dashed purple). **(B)** Light via glutamatergic signaling leads to calcium entry and activation of CREB leading to the transcription of the *period* genes within the SCN core during the night. **(C)** The SCN sends excitatory glutamatergic signals to the PVN during the night which activates melatonin synthesis and release within the pineal gland. During the day, the SCN sends inhibitory GABA inhibit melatonin release. CRH from the PVN also inhibits melatonin via modulation of VIP. RHT, Retinohypothalamic tract; Glu, Glutamate; Opn4, Melanopsin; SCN, Suprachiasmatic Nucleus; VIP, Vasoactive Intestinal Peptide; AVP, Vasopressin; PVN, Paraventricular Nucleus of the Hypothalamus; CRH, Corticotropin Releasing Hormone; ACTH, Adrenocorticotrophic Hormone; CREB, cAMP response element binding protein.

Light exposure allows for the entrainment of circadian rhythm to the 24-h day. Morning light shifts the circadian timekeeping system earlier (phase-advance) and evening light shifts it later (phase-delay). The time at which the circadian system promotes sleep or wake can be influenced by internal factors (endogenous circadian period and circadian photosensitivity) as well as external factors (light exposure). The circadian timing system relies on the photopigment melanopsin (Opn4), rather than the visual photoreceptor rhodopsin, to relay light signals to the suprachiasmatic nucleus (SCN) through intrinsically photosensitive retinal ganglion cells (ipRGCs) in the retinohypothalamic tract. Within the SCN core, vasoactive intestinal peptide (VIP)-containing neurons receive direct retinal inputs, whereas the vasopressin (AVP)-containing neurons within the SCN shell receive GABAergic and VIP input from the core. Coupling the oscillatory network between core and shell generates self-sustaining pacemaker rhythms, with SCN outputs of VIP and GABA being sent to other hypothalamic nuclei, while GABA and AVP are sent as outputs to other parts of the brain (Figure 1A; Barko et al., 2019). Lastly, photic signals to the SCN core can directly affect circadian clock genes expression (e.g., *period*), leading to the determination of the phase of the mammal (Figure 1B).

Connections exist among the hypothalamic–pituitary–adrenal (HPA) axis and the circadian rhythm, both of which can be modulated by light exposure. For normal responses to stressors, corticotropin-releasing hormone (CRH) from paraventricular nucleus of the hypothalamus (PVN) stimulates the pituitary gland to secrete adrenocorticotropic hormone (ACTH), which in turn prompts the adrenal glands to release cortisol (or the rodent equivalent corticosterone/cort; Figure 1A). Cort then binds to glucocorticoid receptors (GRs), which are expressed throughout the body, to regulate glucose metabolism and brain function during times of stress. Chronic stress, regardless of the source, is associated with increased CRH (Chappell et al., 1986). In both diurnal and nocturnal animals under normal lighting conditions, cort levels surge at the start of the active period to promote waking, then drop during the remainder of the day, starting to rise again immediately before the start of the active cycle. Recent studies illustrate that VIP-containing SCN-core neurons directly innervate the PVN and that VIP from the SCN is necessary to convey light cues to the PVN to regulate the HPA axis. Ablation of molecular clock in the PVN leads to desynchronization of CRH release (Jones et al., 2021). Silencing VIP during the early day increases corticosterone release later in the subjective day (Paul et al., 2020). A second pathway exists involving Neuromendin S (NMS) from the SCN to dopaminergic neurons in the PVN which can also regulate CRH levels. SCN NMS is upregulated under short-day photoperiods (<5 h), which activates dopaminergic PVN neurons and inhibits CRH production (Porcu et al., 2022). As such, the role of the SCN is to inhibit HPA axis activation after the cort surge at the start of the active period through the synchronization of PVN “core clock gene” rhythms, inhibiting CRH-producing neurons.

Under standard light:dark cycles, inhibitory and stimulatory signals from the SCN regulate the pineal gland so that melatonin is produced during the night. Melatonin is produced via SCN innervation of the PVN, initiating norepinephrine release, which then activates the pineal gland to synthesize melatonin. GABAergic signals from the SCN inhibit melatonin release from the pineal gland during the day via the PVN (Kalsbeek et al., 2000). At night, glutamatergic signaling from the SCN leads to melatonin synthesis and release (Perreau-Lenz et al., 2004). Therefore, the SCN uses GABAergic daytime inhibitory signals and nighttime glutamatergic stimulatory signals on the PVN-pineal pathway – GABA responsible for the (daytime) inhibitory signal and glutamate for the (nighttime) stimulation and release of melatonin (Figure 1C). Melatonin also has a role in inhibiting the CRH-induced release of ACTH and cortisol (Tsukamoto et al., 2013), so when melatonin levels are low during morning light, the HPA axis can exert its effects. In turn, CRH from the PVN inhibits melatonin release from the pineal gland (Kellner et al., 1997) and stimulation of the PVN and CRH by GABAergic neurons in the SCN core promotes wakefulness (Ono et al., 2021).

Regardless of age, light-at-night increases both CRH and cort levels and the number of cells that produce them (Milosević et al., 2005; Dulcis et al., 2013). Even short-term exposure to light-at-night (0.5–3 h) can lead to increased anxiety due to increased GRs and cort (Loh et al., 2008; Wang et al., 2023). Recent research also illustrates that the mood-altering effects of light-at-night are mediated through Opn4 inputs to the amygdala, the perihabenula nucleus of the thalamus, and prefrontal cortex (PFC; Fernandez et al., 2018; Li et al., 2024a; Lazzerini Ospri et al., 2024). Additionally, individuals with mood disorders exhibit reduced nighttime melatonin secretion (Li et al., 2024b). In summary, research suggests a mechanism whereby: (1) the circadian timing system regulates the release of CRH from the PVN (and subsequently ACTH and cort) through photic signaling pathways, (2) evening light suppresses melatonin, which can stimulate nighttime HPA activation to promote wakefulness during the inactive phase, and (3) that exposure to light-at-night can lead to abnormal emotionality due to changes in the HPA axis, melatonin secretion, and the function of brain areas associated with mood regulation.

### 1.2 Adolescents have increased sensitivity to light-at-night

Adolescents are particularly prone to mood disruptions and increased stress due to light-at-night for several reasons related to their developmental stage and behavioral patterns. Evening light (both standard room-level light and dim-light through screentime) has greater delaying and suppression effects on melatonin in adolescents, indicating greater sensitivity to light-at-night compared to adults (Higuchi et al., 2014; Crowley et al., 2015; Figueiro and Overington, 2016). Melatonin suppression is correlated with increased prevalence and exacerbated symptoms of psychiatric disorders, which tend to present during adolescence, including bipolar disorder and depression (Chauhan et al., 2023). In animal models, exposure to light-at-night prior to adolescence can result in anxiety-like and depressive-like behaviors during adulthood, even when the evening light was removed (Borniger et al., 2014; Cissé et al., 2016; Chen et al., 2021). Adolescent exposure to light-at-night also leads to poorer memory, increased anxiety, and activation of the amygdala (Bonilla et al., 2024). In addition to altered melatonin secretion and sensitivity to light-at-night, teenagers also exhibit altered phase and levels of cortisol, particularly during the morning surge to promote wakefulness (Carpenter et al., 2015). Adolescent girls with conduct disorder exhibit reduced morning cortisol levels compared to girls without conduct disorder, indicating a reduced or phase-delayed HPA axis (Helleman et al., 2023). Stressors also produce reduced morning cortisol secretion and dampened HPA axis function in response to aberrant light exposure in both teenagers (Chiang et al., 2016) and juvenile mice (Miller et al., 2022). These results underscore the heightened vulnerability of adolescents to the adverse impacts of light-at-night on the regulation of the HPA axis, making them particularly susceptible to its effects in altering emotionality.

There is a propensity towards evening chronotypes in adolescence partially due to hormonally-driven changes in circadian rhythmicity, which is aggravated by a further delay in wakefulness due to their increased sensitivity to exposure to light-at-night (Hagenauer and Lee, 2013). In particular, sex hormone increases during puberty is associated with increased evening preference in teenagers of both sexes, which is correlated with impaired mood (Dolsen et al., 2019). This change is driven by the timing of melatonin release (even if good light-hygiene is practiced), which occurs later in the evening in teenagers compared to pre-adolescence and adulthood, leading to delays in the timing of sleep–wake cycles, making adolescents feel more alert and less sleepy at night (Carskadon et al., 2004). Teenagers with rhythms delayed toward eveningness exhibit shorter melatonin secretion periods and increased risk of developing mood disorders (Jääkallio et al., 2024). These studies all illustrate that adolescents exhibit an endogenous shifting towards eveningness compared to both younger children and adults, making them more likely to be exposed to light-at-night as well as being more sensitive to its negative effects.

Compounding the tendency for evening chronotypes, teenagers tend to stay awake later and sleep-in on the weekends, when free from mandatory school schedules, leading to further circadian desynchronization in a way circadian scientists call “social jet-lag.” One study reported that teenagers had a school night bedtime approximately 22:30, but during the weekends it was delayed to after midnight with longer sleep; this alteration to the time and duration of sleep was associated with increased anxiety and depression (Zhang et al., 2017). The use of electronic devices during the night influences adolescent sleep timing and quality by inducing circadian phase-delays and exacerbating social jet-lag (Lemola et al., 2015; Hena and Garmy, 2020). Teenagers with mood disorders are especially prone to the adverse effects of light-at-night, as a recent study reported increased likelihood of shorter sleep duration and social jet-lag in clinically-depressed adolescents compared to individuals without depression (Tonon et al., 2022). The cumulative effect of these disruptions – altered melatonin levels, increased cortisol, and impaired emotional regulation – leads to chronic stress, further exacerbating sleep issues and leading to poor mental health.

### 1.3 Novel understanding of how light-at-night negatively affects mood in adolescence

Sleep and circadian disruption through exposure to light-at-night are associated with alterations in brain chemistry and structure, which can lead to abnormal behaviors. Variability in sleeping patterns, including social jet-lag, is associated with reduced white matter integrity within the superior longitudinal fasciculus and posterior thalamatic radiation, areas that modulate emotionality (Telzer et al., 2015; Uy et al., 2023). White matter integrity changes have been observed in people with mood disorders, including depression (Uy et al., 2023). One possible reason for the reduced white matter integrity found in individuals with social jet-lag, mood disorders, and sleep issues through alterations in Brain-derived Neurotrophic Factor (BDNF). BDNF is known to have neuroprotective effects to white matter integrity (Husson et al., 2005; Weinstock-Guttman et al., 2007) and evidence illustrates that anti-psychotic drugs and antidepressants can ameliorate white matter lesions and increase myelination through the activation of BDNF pathways (Xiao et al., 2010; Sun et al., 2022). Meanwhile, light-at-night has been shown to reduce BDNF levels within the PFC (Capri et al., 2019), while increasing BDNF within the amygdala (Li et al., 2024a). As adolescence is a crucial period for brain development, particularly in areas involved in emotional regulation, light-at-night can impair the development and functioning of these regions, making adolescents more vulnerable to developing mood disorders.

Stressors can create increased inflammatory responses within the amygdala and PFC, which is associated with white matter lesions and mood disorders in adolescents (Thomas et al., 2021; Doney et al., 2022; Poletti et al., 2022). In rodents, light-at-night is known to produce increases to inflammatory cytokines in the amygdala and PFC, which is positively correlated with the intensity of behavioral issues (Walker et al., 2020; Kumari et al., 2021; Jerigova et al., 2023). Increased BDNF is associated with reduced inflammation in individuals with depression (Zhang et al., 2016), while reduced BDNF and increased inflammation are associated with exacerbated symptoms of mood disorders in adolescents (Karthikeyan et al., 2022). Therefore, reducing the amount of inflammation may be a method to alleviate some of the negative behavioral outcomes associated with mood disorders.

**Figure 2:**
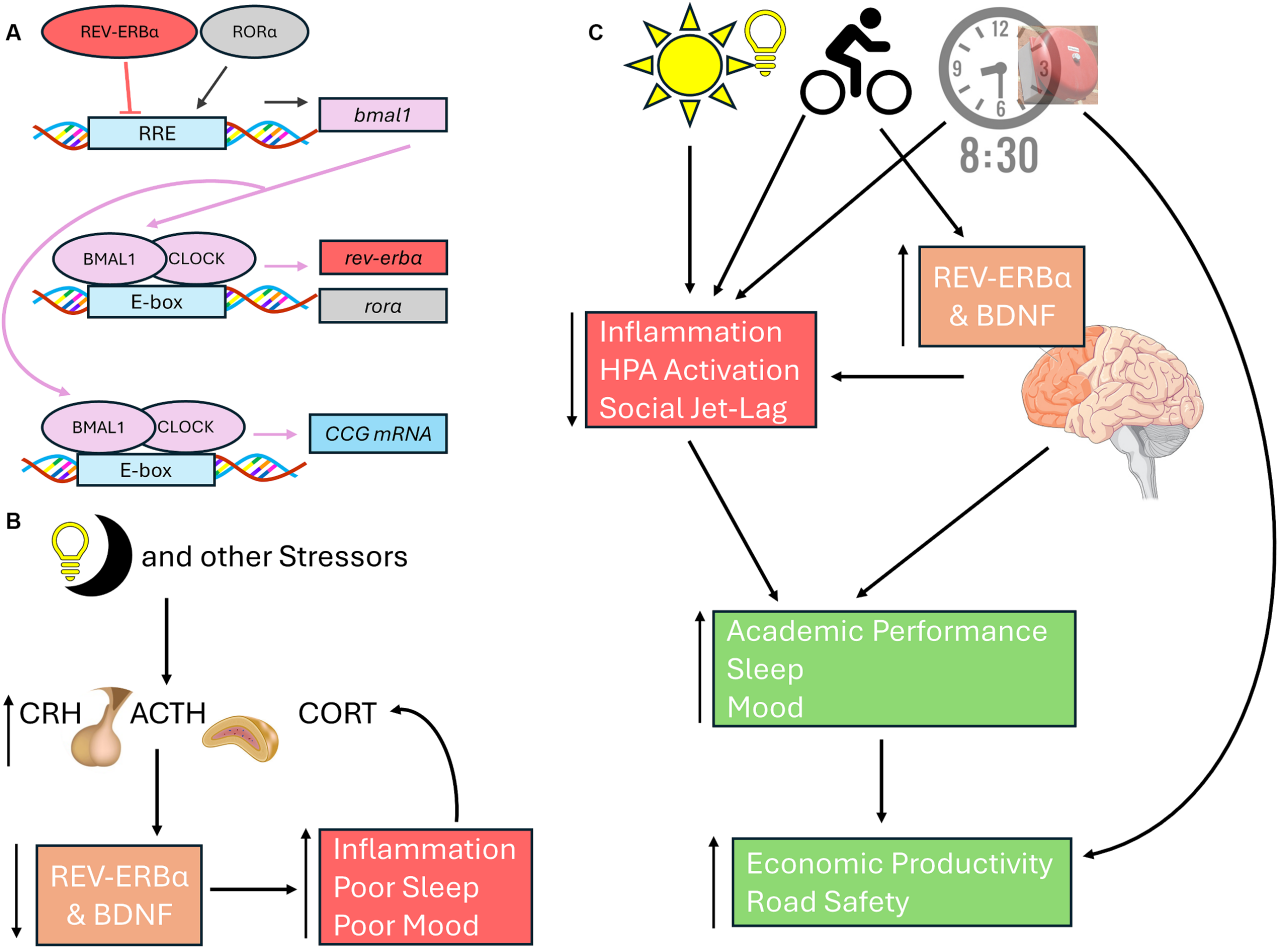
Connections among neuronal health, *rev-erbα*, and light-at-night. **(A)** In the positive loop of the molecular clock under standard light:dark cycles, BMAL1 and CLOCK heterodimers bind to the E-boxes of the *rev-erbα* and *rorα* to promote their transcription. RORα induces *bmal1* transcription, REV-ERBα represses *bmal1* expression through activating or inhibiting RREs, respectively. Once translated, BMAL1 can form heterodimers with CLOCK to promote the transcription of CCGs, including *period*. **(B)** Light-at-night, HPA axis activation, and other stressors inhibit BDNF and *rev-erbα*, leading to poorer sleep, inflammation, and mood issues, each of which in turn can further exacerbate all three issues. **(C)** Exposure to morning light, high school start times of 8:30 am or later, and morning exercise leads to improved emotionality in adolescents through better sleep and reduced social jet-lag, reduced stress, and better academic performance. Both morning light and exercise increases and stabilizes BDNF and *rev-erbα*, leading to improved neuronal health in areas associated with mood regulation, including PFC. Exercise, BDNF and *rev-erbα* rhythmicity, are all positively correlated cognitive performance. Later school start times improves academic achievement, road safety, and economic outcomes for both students and their communities. Basic Helix-Loop-Helix ARNT Like 1 (BMAL1 also known as ARNTL); CLOCK, circadian locomotor output cycles kaput; REV-ERBα (also known as nuclear receptor subfamily 1 group D member 1 or NR1D1); RAR-related orphan receptor alpha (RORα); CCG, Core Clock Genes; BDNF, brain derived neurotrophic factor; PFC, Prefrontal cortex.

While numerous studies have linked the negative loop of the molecular clock (i.e., the BMAL/CLOCK regulation of *period* – Figure 1B) to mood disorders, recent work is linking the effects of light-at-night on the positive loop (regulation of BMAL1 via *rev-erbα* and *rorα* – Figure 2A) on mood and stress responses. Light-at-night decreases and alters rhythmicity of *rev-erbα* expression not only within the SCN, but also within areas that control emotionality, including other hypothalamic nuclei, the PFC, and the amygdala (Cissé et al., 2016; Otsuka et al., 2020). Part of the reason for this impaired emotionality seen in individuals exposed to light-at-night is due to the desynchronization of expression patterns of core clock mRNA and protein levels between the SCN and other brain areas, including the amygdala (Ikeno and Yan, 2016; Bonilla et al., 2024). Additionally, both *rev-erbα* knockout mice and knock-down of *rev-erbα* are associated with increased anxiety-like, and depression-like behaviors, through modulation of dopaminergic and serotonergic signaling, neurotransmitter pathways associated with emotional health (Chung et al., 2014; Zhao and Gammie, 2018; Otsuka et al., 2022; Chen et al., 2023). REV-ERB agonists also have anxiolytic effects (Banerjee et al., 2014). Connections exist between *rev-erbα* expression and the HPA axis, wherein GR agonists and stressors suppress *rev-erbα* expression (Murayama et al., 2019). Lastly, *rev-erbα* and inflammation are also connected as reductions in *rev-erbα* lead to increased inflammatory responses, while *rev-erbα* itself can reduce inflammation (Sato et al., 2014; Figure 2B). Although these aforementioned studies provide good evidence for the potential therapeutic effects of targeting *rev-erbα*, very few studies have investigated the role of the positive loop of the molecular clock on the development of mood disorders and stress responses in adolescents, emphasizing the urgency of addressing this gap of knowledge specifically within this age group.

## 2 Discussion and conclusion

The first step to mitigating these issues is to reduce light exposure during the night. One study indicated that evening chronotype in adolescents is less severe in rural locations where there is less exposure to artificial light-at-night (Vollmer et al., 2012). Reducing light-at-night is not as simple as it sounds, as adolescents face various psychosocial challenges, including academic pressures, social interactions, and athletic competitions which promote a trend toward eveningness. The best way to improve sleep and cognitive performance in teenagers during the day is to stop using screens after 9 pm (Perrault et al., 2019). Additionally, schools can add the biological and health consequences of light-at-night exposure to the curriculum, as preliminary evidence supports that students would be interested in pursuing methods that reduce light pollution (Yüzbaşıoğlu et al., 2020).

Early school start times force adolescents to wake up earlier than their biological clocks dictate, leading to sleep deprivation, social jet-lag, and stress. School start times of 8:30 am or later lead to improved academic performance, less sleepiness, and improved mood in high school students (Yip et al., 2022; Yeo et al., 2023). Longer school commute time is associated with increased HPA axis activation, particularly in adolescents with evening chronotypes (Karan et al., 2021). Additionally, later school start times can contribute to improved road safety, especially for adolescents (Bin-Hasan et al., 2020). Advocating for policy changes by involving key stakeholders, including parents, teachers, school administrators, and students, in the decision-making process and sharing success stories from other school districts that have implemented later high school start times and experienced positive outcomes could lead to this change (Watson et al., 2017). Delaying school start times also have positive economic impacts for both communities and students’ future salaries (Hafner et al., 2017). Though it may pose logistical challenges including interfering with extracurricular activities, transportation, and misaligning with parental schedules, solutions could be discussed to help convince stakeholders of the importance of making this change for adolescent well-being and success.

If school times were to start later, adolescents will have to resist the urge to stay-up later, thinking that they can compensate the later school day with a later bedtime, necessitating a discussion about the importance of good sleep hygiene with teenagers. Poor sleep hygiene is associated with mood disorders in adolescents (Short et al., 2020). One method in promoting sleep hygiene among adolescents is for caregivers to set an example by practicing healthy sleep habits themselves (Brand et al., 2009). Exposure to daytime light can help promote better sleep at night and lead to a phase-advance that can prevent social jet-lag, and impairments in mood (Misiunaite et al., 2020). Low-intensity exercise during the morning can also lead to phase-advances and improvements in mood, while sedentary lifestyles can exacerbate evening chronotypes in adolescents (Lang et al., 2022). Exercise is associated with increases in BDNF and *rev-erbα*, enhanced sleep, and reduced inflammation, leading to improved mood (Brand et al., 2010; Dopp et al., 2012; Wunram et al., 2021; da Rocha et al., 2022; Figure 2C).

In conclusion, the relationship among adolescent mood, stress, and light-at-night, highlights the critical importance of ensuring proper sleep, health, and well-being for this age group. While the precise anatomic links among the HPA axis, the biological clock, and photic pathways are known, these studies have been conducted predominately using adult animal models, highlighting the need for additional preclinical studies which investigate the effects of aberrant light exposure on adolescent development. Reducing light pollution, particularly exposure to artificial light-at-night, plays a vital role in promoting healthy sleep patterns as adolescents are more sensitive to it. By implementing these strategies, adolescents can minimize their exposure to light-at-night and promote healthier sleep patterns and reductions in stress.

## Author contributions

GG: Conceptualization, Writing – original draft, Writing – review & editing. CM: Conceptualization, Writing – original draft. MM: Conceptualization, Writing – review & editing. JS: Conceptualization, Supervision, Writing – original draft, Writing – review & editing.
